# Lisbon’s COVID 19 response: harm reduction interventions for people who use alcohol and other drugs in emergency shelters

**DOI:** 10.1186/s12954-021-00463-x

**Published:** 2021-01-25

**Authors:** Ricardo Fuertes, Elsa Belo, Cristiana Merendeiro, Adriana Curado, Diana Gautier, Ana Neto, Hannah Taylor

**Affiliations:** 1 Municipality of Lisbon, Lisbon, Portugal; 2 Ares do Pinhal, Lisbon, Portugal; 3 Crescer, Lisbon, Portugal; 4 Grupo de Ativistas em Tratamentos, Lisbon, Portugal; 5 Médicos do Mundo, Lisbon, Portugal; 6 Unidade de Alcoologia de Lisboa, Lisbon, Portugal

**Keywords:** Harm reduction, Alcohol, Drugs, Homelessness, Emergency shelters, COVID-19

## Abstract

Four emergency shelters were instituted in Lisbon during COVID-19, and are still in operation. Between March and August 2020, they served over 600 people. The shelters host a diverse population, including people experiencing homelessness, foreigners, LGBTI + people, those with reduced mobility, couples, those with pets, and People Who Use Drugs, including alcohol (henceforth PWUD). Individuals are provided care regardless of their immigration or residence status. In order to ensure continuity of care in the shelters and to bring in clients who usually refuse to be sheltered, a range of social and health interventions are integrated into the shelters. Harm reduction services ensure that the most vulnerable populations, PWUD and people experiencing homelessness, have access to the services they need. Innovations in service provision maximize the services impacts and pave the way for the future inclusion and development of these services.

## Background

COVID-19 arrived in Portugal later than in neighbouring countries, with the first  known cases on March 2, 2020. As in other countries, there was a sense of uncertainty before it was clear how confinement measures would be implemented. For clients of Lisbon’s harm reduction and treatment services, and those providing these services, the panic was more acute. Lisbon has a robust network of harm reduction services including street teams, needle and syringe programmes, Opioid Substitution Therapy (OST) provision and a mobile drug consumption room (MDCR). Whether people would be able to move freely, and how services would be able to maintain operation, were pressing questions.

Preparing for the possibility of mandatory confinement, the municipality of Lisbon created emergency shelters. The first shelter opened on March 16, three days before the State of Emergency was declared. Three more shelters opened, in addition to five for those who tested positive for COVID-19 who did not need to be hospitalized but did not have a place with proper conditions to stay.

The four shelters for people who were not infected with COVID-19 are still in operation as of today and serve a diverse population. Some are chronically homeless, others lost their work or housing as a result of the economic effects of COVID-19, and others were unable to return to their home countries due to the pandemic. The shelters' combined capacity is 220 people.

People Who Use Drugs (PWUD) and people experiencing homelessness face the same COVID-19-related health risks as does anyone during the pandemic, as well as additional risks. Many PWUD have comorbid health conditions that can predispose them to COVID-19-related complications [1]. Certain substances and ways of using substances aggravate health conditions that make PWUD more susceptible to COVID-19-related complications, through stressing the pulmonary system, depressing the immune system or causing cardiovascular issues [5].

In general, marginalized populations tend to have poorer health conditions and face additional barriers, such as stigma, to accessing care [4]. Being told to ‘stay at home’ simply does not work for everyone, especially not people experiencing homelessness [4]. They may not have an adequate and sanitary place to stay and be cut off from services, such as access to food [2] or OST. With less people on the streets, ways to make money like panhandling or recycling become unavailable [5]. Emergency financial relief measures can exclude those who make money through the informal economy, which can force individuals into even more dangerous situations, such as risky sex work [4]. Marginalization can also lead to lack of access to information concerning the virus [4].

In the case of PWUD, forced social isolation can be difficult to accomplish and even increase risks. Many behaviours associated with drug use, such as congregation and material sharing, place PWUD at a higher risk for COVID-19 exposure [1]. Being forced into isolation increases the risks associated with consuming alone, such as being unable to seek help in case of overdose. Additionally, PWUD are at a higher risk for mental health conditions [1] which can be exacerbated by isolation and lockdown.

In the context of confinement, purchasing drugs can become much more difficult, as movement is restricted. Personal use patterns may also shift. For example, during the COVID-19 pandemic there has been an increased consumption of alcohol and benzodiazepines [1]. Effects on the drug market may include higher prices, reduced availability, and sometimes reduced purity [1]. Temporary interruptions of heroin availability have been seen in Europe, along with changes in the availability and price of cannabis in Portugal [1].

There is evidence of adaptability and innovation in health and social service provision during the pandemic. That has meant, in some contexts, allowing more take-home OST doses or broadening access to naloxone in the community [1]. There has also been evidence of increased uptake of services, such as OST [1], as people recognized potential interruptions to their supplies of drugs, income, or harm reduction supplies.

In responding to the vulnerabilities facing already-marginalized populations, it is extremely important to ensure the continuity of care [6] in all services that clients utilize, as well as to give access to new services which they may require. During the pandemic, it was determined to be more effective and safer for the network of health and social services to approach shelter residents in Lisbon, not the other way around.

## Description of model or intervention

The responses integrated in Lisbon’s emergency shelters were not created during the pandemic; they were pre-existing, well-developed interventions in the city. The teams involved already had long-standing relationships of trust with some clients from the populations of PWUD and those experiencing homelessness. The city council and national health service collaborated with those pre-existing interventions, delivered by governmental and non-governmental organizations (NGOs). All teams worked collaboratively and in coordination with the centres' management. Whenever possible, interventions were carried out in or near the shelters (Table 1, 2, 3).

**Table 1:** Health screening and tests

Service	Providers	Uptake, when known	Results
HIV rapid testing	NGOs GAT/Médicos do Mundo	111 tests (Apr–July)	4 reactive tests
HCV rapid testing	NGOs GAT/Médicos do Mundo	89 tests (Apr–July)	16 reactive tests
HBV rapid testing	NGOs GAT/Médicos do Mundo	89 tests (Apr–July)	2 reactive tests
Syphilis rapid testing	NGOs GAT/Médicos do Mundo	104 tests (Apr–July)	5 reactive tests
TB screening (chest x-ray)	NHS: Centro de Diagnóstico Pneumológico	238 people	1 infection
COVID testing	NHS	100 tests (clients and staff) (March–July)	1 infection

**Table 2:** Harm reduction interventions

Service	Providers	Uptake, when known
Mobile drug consumption room	NGOs GAT/Médicos do Mundo	22 people 10 visits daily
Needle and syringe program/crack pipes	NGOs Crescer/GAT/Médicos do Mundo	4029 needles/syringes 155 crack pipes
Training in overdose response, nasal naloxone distribution	NGOs GAT/Médicos do Mundo	63 people trained (staff and clients)
Treatment to prevent alcohol withdrawal syndrome (Diazepam (10 mg); Thiamine, Folate and Tiapride (100 mg), 2 × daily)	NHS: Unidade de Alcoologia de Lisboa	22 people

**Table 3:** Other social and health interventions

Service	Providing entity	Uptake, when known
Psychiatric support	NHS: Centro Hospitalar Psiquiátrico de Lisboa, Centro Hospitalar Lisboa Norte, Centro Hospitalar Lisboa Oriental, Centro Hospitalar Lisboa Central	170 appointments, 100 people between March and 25th June
Social support and accompaniments to social services and medical appointments	NGOs coordinated by Ares do Pinhal	102 referrals to state social support 45 referrals to the hospital 18 referrals to embassies 7 transportations to other cities (March–June)
Support for employment	NGO Rede Emprega	95 people 51 looking for a job 44 working (March–June)
Housing First project for people who experience chronic homelessness and use drugs	NGO Crescer	54 referred 23 integrated in individual houses 8 waiting for integration (March–June)

In adjusting to the present circumstances, sanitation and physical distancing were integrated into each team’s efforts in order to minimize the spread of COVID-19. Teams adjusted their procedures by reducing contact with clients in closed settings, wearing masks, and working in shifts. The residents of the centres were monitored daily for any signs of infection or symptoms. About 100 COVID tests were performed on staff and clients at the shelters. There was one positive case in one shelter, and one positive case identified during admission and referred to the hospital.

As the streets emptied, drug consumption became more visible during the pandemic, leading to friction between PWUD and other residents. The largest centre, housing 90 people, is in a residential area. There were complaints from the community members there, via phone calls, emails, and Facebook groups. They asked for increased availability of the MDCR, increased police presence and to create several small shelters rather than this large one. This Not in My Backyard (NIMBY) effect was counteracted by establishing direct contact with residents, by organizing a daily clean-up by residents and staff, and by cleaning away vegetation which had become a site of increased drug use. The daily clean-up facilitated a positive relationship with the neighbourhood not only through addressing the problem of discarded waste, but also through increased communication. A new stop of the MDCR was also added in a park near the shelter.

A sense of participation and community involvement was maintained in the shelters through various means. About every 2 weeks, there were assemblies where residents could discuss problems and devise solutions, which were voted on. Most of the harm reduction teams in Lisbon have peer workers as a part of their staff, so the methadone, needle exchange, and MDCR teams brought peer involvement into the shelters. Peer integration facilitates trust and competence in service provision and helps new clients to approach the services.

There were examples of innovations and fast progress during this period. Previously, DCR access and naloxone training had not existed in shelters in Lisbon. The low-threshold alcohol treatment represented a new intervention in Lisbon, in that it could be administered on demand, by a nurse, without a medical evaluation. Of the 22 people who utilized this service, most of them indicated interest in a structured alcohol treatment. Five of them have so far been clinically assessed and linked to the Alcohol Treatment Unit. 

Many people who stayed at the shelters at some point were transitioned into medium to long-term housing. Lisbon has a municipal plan which aims to house all people who are experiencing homelessness, primarily through Housing First programs. Housing First provides people with an immediate and permanent house combined with support, without requiring psychiatric treatment or sobriety. The program accelerated during COVID-19, and 300 houses were made available, in addition to the pre-existing 80. The primary target beneficiaries were PWUD, and those with mental health conditions or other forms of vulnerability. So far, 54 PWUD were referred to Housing First programs, 23 of whom were integrated and 8 of whom are in the process of benefiting from it.

## Conclusions

Between March and August 2020, over 600 people were hosted in Lisbon’s emergency shelters. The design of the emergency shelters was inclusive of different identities and needs. The integrated design allowed for access to a wide variety of necessary services. Community integration, both within the shelter and between each shelter and its surroundings, was prioritized and achieved through mechanisms of participation, self-governance and open communication.

Some of the services that were provided were the first of their kind, or the first in a shelter setting. The availability of these services brought in some clients who usually refuse to be referred to a shelter. In particular, the services designed for active drug users opened up the discussion of making these services available even after the pandemic. In April, for example, the Portuguese National Drugs Agency (SICAD) announced an intention to open up a managed alcohol program for people who use alcohol [3].

## Data Availability

Information about the interventions is available upon request.

## Abbreviations

AUD: Alcohol Use Disorders
ARSLVT – UAL: Administração Regional de Saúde de Lisboa e Vale do Tejo - Unidade de Alcoologia de Lisboa
COVID-19: Corona Virus Disease 2019
MDCR: Mobile Drug Consumption Room
NGOs: Non-governmental organizations
PWUD: People Who Use Drugs
OST: Opioid Substitution Therapy
SICAD: Serviço de Intervenção nos Comportamentos Aditivos e nas Dependências
